# Reimagining information literacy instruction in an evidence-based practice nursing course for undergraduate students

**DOI:** 10.5195/jmla.2019.663

**Published:** 2019-10-01

**Authors:** Bethany Sheriese McGowan

**Affiliations:** Assistant Professor of Library Science and Health Sciences Information Specialist, Library of Engineering and Science, Purdue University, West Lafayette, IN, bmcgowa@purdue.edu

## Abstract

This case report describes the redesign process for an undergraduate evidence-based practice (EBP) nursing course in which the librarian serves as both co-instructor and co-instructional designer. As part of the undergraduate outcomes-based core curriculum, this required course teaches the principles of the research process; teaches students to identify the strengths and limitations of research articles in relation to EBP; and builds student confidence in their abilities to execute information literacy, data management, and scholarly communication competencies. The course redesign built on an existing student-centered course design, with the specific goal of transitioning the course from a senior-level course to a sophomore-level course, while achieving the same learning objectives. This goal was accomplished by integrating a combination of distributed practice and interleaved practice learning experiences into the course curriculum.

## BACKGROUND

This case report describes the redesign process for an undergraduate evidence-based practice (EBP) nursing course, for which the nursing liaison librarian has served as both co-instructor and co-instructional designer. The course is part of the undergraduate, outcomes-based core curriculum and teaches sophomore nursing undergraduates the principles of the research process; teaches students to identify the strengths and limitations of research articles in relation to EBP; and builds student confidence in their abilities to execute competencies in information literacy, data management, and scholarly communication. Course enrollment typically ranges between sixty and sixty-five students per semester, and the course is offered in both the spring and fall semesters.

Six years prior to the redesign detailed in this report, the course had undergone a redesign in the IMPACT Program. IMPACT, a campus-wide initiative [1], aims to incorporate student-centered teaching practices and learning technologies into courses [2, 3]. Faculty work with a support team of instructors from the libraries, the Center for Instructional Excellence, and campus Information Technology: Teaching and Learning Technologies. In courses that have gone through IMPACT redesigns, faculty report significant increases in student activity and engagement and perceive an improvement in students’ critical thinking skills [2]. The nursing liaison librarian had been co-teaching the course for one academic year. Prior to her involvement, the previous nursing liaison librarian was involved with the course and advised on its initial IMPACT redesign.

After the undergraduate EBP nursing course’s initial IMPACT redesign, a curriculum shift occurred. The information literacy–based competencies taught in the course were considered better suited for sophomores, who are just beginning research projects, as opposed to seniors, who are preparing to graduate. Though the course would be taken by less-experienced students, course learning objectives were set in accordance with accreditation standards and remained fixed. These objectives were numerous and wide-ranging:

1. describe the principles of research and the process of EBP;
2. use information and information technologies ethically, legally, and proficiently;
3. explain the purpose and methodology of various types of quantitative and qualitative research designs;
4. evaluate the quality of research evidence to determine scientific merit, strengths, and limitations relevant to clinical practice;
5. examine the economic, legal, and ethical issues related to conducting research;
6. discuss the process of translating research evidence into practice; and
7. demonstrate the characteristics of an innovator that are necessary for EBP, including leadership, a sense of inquiry, flexibility to change, awareness of self and the environment, effective communication, critical thinking, lifelong learning, and professionalism [4].

The original course curriculum assumed that students had already mastered basic information literacy competencies, and while appropriate for an audience of seniors, that assumption did not hold true for sophomores. Another redesign was needed to achieve all the aforementioned course learning objectives using activities and assessments that were appropriate for sophomore-level undergraduates.

Additionally, the faculty and librarian co-instructors observed a decrease in the sophomore students’ engagement in the course. We hypothesized that this was because many students failed to complete the required readings and other nongraded prework, possibly because they concurrently took other difficult courses and the heavy course load challenged their time-management and study skills. This was especially evident as the student-focused course design required students to lead and propel course discussions. Therefore, another redesign objective was to increase student motivation and participation.

The course redesign sought to maintain a student-centered approach. Well-designed student-centered learning approaches motivate students to take an active role in the learning process [5–9]. Findings from cognitive and educational psychology, particularly Deci and Ryan’s self-determination theory and Dunlosky’s review of effective learning techniques, were considered. Deci and Ryan’s self-determination theory provided a motivational framework that advised that learning experiences should be based on the fulfilling of three needs:

1. “Autonomy,” feelings of volition and choice, which are supported by endorsement of behavior and student ownership of the learning process;
2. “Competence,” the extent to which students believe they have mastered content and are able to perform academically; and
3. “Relatedness/Relevance,” feelings of belongingness and connectedness with others and with the material presented in class [10].

Educational psychologist Dunlosky tested ten learning techniques and concluded that practice testing (i.e., self-testing) and distributed practice (i.e., implementing a schedule of practice that spreads out study activities over time) are both highly effective learning techniques. His findings also suggest that interleaved practice (i.e., implementing a schedule of practice that mixes different kinds of problems and materials in one study session) is particularly effective for math and concept learning. These time-saving techniques could prove especially useful for sophomore students who are still establishing study skills. For example, findings from Dunlosky’s research suggest that the popular approach of rereading material is inefficient and that time would be better spent practice testing [11].

Together, these theories helped form the framework for a course redesign that provides motivation for learning and equips students with effective learning practices.

## CASE PRESENTATION

The Blended Librarians Adapted ADDIE Model (BLAAM), Bell and Shank’s instructional systems design process adapted for academic librarians, was used to plan the course redesign. BLAAM consists of five phases: “Assess,” “Objectives,” “Develop,” “Deliver,” and “Measure” [12].

### Assess

The Assess phase involved gaining an understanding of the needs of the current environment, including an assessment of course outcomes and student deficiencies. During this phase, the nursing liaison librarian discussed learning outcomes with the faculty instructor and conducted an informal needs assessment via reference interviews and similar structured conversations with students to better understand their challenges and needs. The deliverable for this phase was a problem statement: “Student engagement and participation performance during class suggests that students are not completing assigned readings and prework activities.”

### Objectives

The Objectives phase established the process for what the instruction design needs to accomplish to be considered successful. Bell and Shank suggest an “Audience-Behavior-Condition-Degree” (A-B-C-D) process for developing design objectives [12]. For this redesign, the audience was sixty-two sophomore-level nursing students in a four-credit, in-person course. The behavior was increased student engagement and participation. The condition was that the learner related the knowledge that they gained from completing prework assignments to actively engage in class activities, ultimately building confidence in their ability to execute information literacy competencies. The standard for determining the degree to which the learner achieved the objective was based on instructional observation (i.e., instructors’ perception of increased engagement during in-class activities) and assessment performance.

The objective of the course redesign was to create a learning experience that meets course objectives and encourages student motivation and participation by providing autonomy, competence, and relatedness. We aimed to encourage students to adopt effective learning techniques by introducing course readings and prework assignments that followed an interleaved practice, mixing different kinds of problems and materials in weekly assignments. Also, we wanted to improve student participation and engagement during in-class group activities as measured by qualitative and quantitative assessments, including instructors’ observations of student performance during group work and the response rates of low-stakes, in-class quizzes. Course modules followed a distributed practice, spreading out study activities over time and iteratively building on information literacy competencies.

### Develop

The Develop phase focused on the production process for creating an instruction service using a four-step model: “Prototype,” “Create/Build,” “Formative Evaluation,” and “Revision” [12]. In the Prototype step, we designed a mock-up of a sample course flow. Newly redesigned course assignments—including pre-assignments, in-class assignments, and post-class assignments or homework—followed an interleaved practice approach. This mixture of problems and materials included reading assignments that required students to write summarizations of to-be-learned information; use discussion boards and social media outlets to pose and respond to a “Why” question (elaborative interrogation approach); and use self-paced modules and videos accompanied by quiz questions that required self-explanation of how newly acquired information related to previously known information.

In the Create/Build step, we decided what materials to produce and what types of media to include. The development of new activities and assessments was the heart of the course redesign. The new course approach introduced basic information literacy competencies and then built on them, allowing students to think critically about how the information literacy competencies related to the course objectives. Supplemental Appendix A provides an example storyboard for an in-class search strategy building activity.

Useful tools and media for newly launched activities included Solstice, a wireless content sharing technology for classrooms that was already available at the university; HotSeat, a home-grown application allowing learners to screencast questions and comments in class using short message service (SMS) texting, iOS and Android devices, and desktop and mobile websites; Twitter; the JoVe video library, for which the library already held a subscription; NurseLogic 2.0 web-based interactive tutorials that guide students through foundational concepts; and ThePoint, a digital textbook companion that includes practice quizzes, interactive flashcards, and case studies. The course was also moved to an active-learning classroom that better supported group work and use of Solstice and HotSeat. Supplemental Appendix B details a full course schedule with examples of the revised course flow and the redesigned assignments and assessments.

In the Formative Evaluation step, we tested draft versions of the proposed activities on graduate and undergraduate research assistants, librarians, and discipline faculty members.

In the Revision step, we used feedback from the formative evaluation to finalize the redesigned course schedule and course assignments. An example of the final version of an in-class search activity was the “Searching as Strategic Exploration Building Block Activity” [13].

### Deliver

The Delivery phase process consisted of three steps: “Diffusion,” “Training,” and “Resource Allocation and Budget.” In the Diffusion step, we developed a plan to adopt the instructional product. In our four-credit-hour course, students meet face-to-face in the classroom for two hours per week and spend an additional two hours per week in self-guided team meetings. During the course, students complete a literature review, a data analysis project, and a professional poster in a mixture of individual and group work. The Blackboard Learning Management System is used to organize and deliver course content.

The course is hosted in an active learning classroom that supports technology-enabled active learning: tables and chairs are mobile; instructors and students can screenshare with Solstice; and Hotseat allows both students and instructors to pose and answer questions. Hotseat can also track which students ask and answer questions, which can be used as a participation measurement. The course’s redesigned approach relies on a distributed practice model and consists of two distinct parts. In part one, students learn about the research process, learn to select and use research-topic appropriate databases, build a literature search based on clearly defined research questions, synthesize findings with clear results and conclusions, and use proper American Psychological Association (APA) formatting. Each of these competencies is delivered iteratively, and they build on each other until students are able to create a carefully crafted research agenda.

In the second half of the course, students put their newly gained skills to work and complete both team and individual research projects. Students learn to use Zotero to manage and share bibliographic data, are introduced to the data management cycle, and complete a data analysis project using Twitter data. Final projects include an oral presentation of an individual research project, an oral presentation based on a group Twitter data project, and a poster presentation based on the group research project and presented at the university’s annual undergraduate research symposium.

In the Training step, we identified the skills needed to execute a successful redesign. Both course instructors were familiar with student-centered instruction as participants of the IMPACT program. Additionally, the librarian instructor was an alumnus of the Association of College & Research Libraries (ACRL) “Immersion Teacher Track Program.” We determined further training on course assessment and qualitative research analysis was needed to effectively measure student learning, so the librarian attended an ACRL “Assessment in Action” workshop.

In the Resource Allocation and Budget step, we determined the funding needed for a successful implementation. Because this redesign built on an existing student-centered course on a campus that was heavily invested in supporting student-centered learning, many useful tools were already available. Still, the redesign project was funded by a small Library Innovation Award, library-sponsored funding to encourage librarians and disciplinary faculty to collaborate and develop new learning experiences. Resource allocations included professional development funding ($1,500 for travel to the American Library Association Annual Conference), supplies ($235 for 5 books on instructional design and research methodology), and technology ($750 for an iPad Pro with keyboard).

### Measure

**Figure 1:**
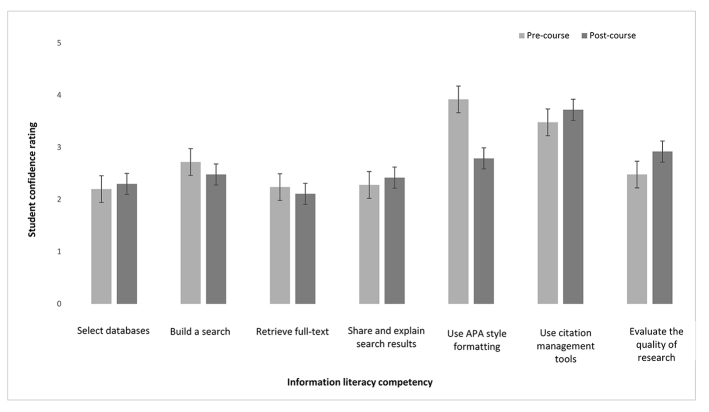
Student confidence ratings in executing information literacy practices before and after completing an evidence-based practice (EBP) course

The Measure phase consisted of pre- and post-course surveys (supplemental Appendix C) that assessed student confidence ratings for several information literacy, data management, and scholarly communication–related competencies. Completion of both surveys was optional, and both surveys were digitally distributed via anonymous links to a Qualtrics survey. Twenty-five students completed the pre-course survey, and 54 students completed the post-course survey. The survey’s Likert items were coded for data analysis: 1=Strongly agree, 2=Agree, 3=Somewhat agree, 4=Neither agree nor disagree, 5=Somewhat disagree, 6=Disagree, 7=Strongly disagree. Differences between pre- and post-course survey responses were analyzed using 2-sample *t*-tests assuming unequal variance. We found significantly increased student confidence, reflected by lower ratings, in using APA style formatting after the course (*t*(41)=3.02, *p*=0.004). All other confidence ratings, however, were unchanged between pre- and post-course surveys (Figure 1).

**Figure 2:**
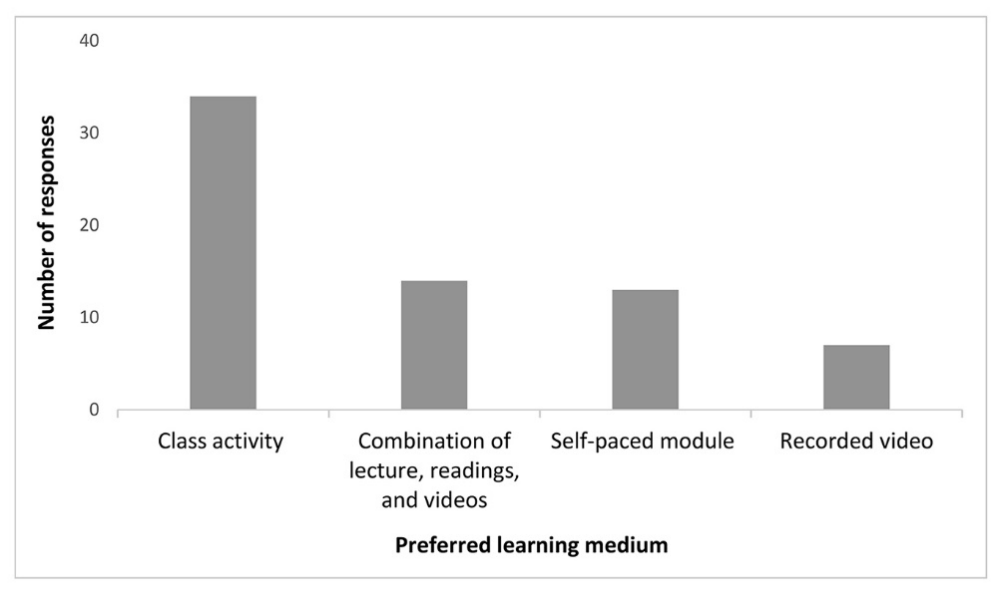
Students’ preferred learning media for information literacy instruction

Subscribing to Marshall McLuhan’s popular phrase, “the medium is the message,” we also sought to understand student preferences for receiving new information. At the end of the course, students were asked to report their preferred learning media, with multiple selections allowed. The overwhelming preferred learning medium was in-class lectures and activities (Figure 2).

## DISCUSSION

We believe the course redesign was a success, because we noted improved student engagement throughout the course, and post-course assessments suggested that students were mostly confident in their abilities to execute information literacy competencies. However, there were no statistically significant gains for most competencies after the course. We speculate that this was because after completing the course, students had a better understanding of the complexities of information literacy competencies and were able to more critically consider their execution. This was best illustrated in the shift in student opinion regarding their ability to evaluate the quality of research evidence, which requires higher order thinking [14] and in which student confidence was lower after completing the course.

We also noted that both assessments were voluntary, and no points were awarded for their completion, which might have produced volunteer bias. Study bias research suggests that participants who volunteer to take part in low-incentive studies tend to have different characteristics from the general population of interest; for example, they tend to be more approval-motivated and have had more robust educational experiences [15]. Despite the mostly null results of statistical analysis in relation to student confidence in executing information literacy competencies, we believe that another indicator of improved student engagement was the marked increase in participation in the post-course survey (87% response rate), compared with the pre-course survey (40% response rate).

The results of the survey of preferred learning media are also interesting. Even after completing a student-centered course that provided a carefully curated selection of learning media—including in-class activities, lectures, readings, videos, and self-paced modules—sophomore students overwhelmingly preferred to learn via in-person lectures and in-class activities. These preferences are common amongst students who are new to flipped classrooms and student-centered learning [16], but using a theoretical framework to inform course redesign (such as our use of Bell’s instructional design model), integrating a mixture of high- and low-stakes assessments into course design, and flipping the entire course and not just selecting activities can help make the adoption of student-centered learning practices more successful and lead to improved student engagement [16].

## SUPPLEMENTAL FILES

Appendix A Storyboard for the design of an in-class search activity

Appendix B Sample course schedule

Appendix C Evidence-based practice course student confidence survey

## 

**Bethany Sheriese McGowan**, bmcgowa@purdue.edu, https://orcid.org/0000-0002-4797-4836, Assistant Professor of Library Science and Health Sciences Information Specialist, Library of Engineering and Science, Purdue University, West Lafayette, IN
