# Supplementary material for: Reimagining information literacy instruction in an evidence-based practice nursing course for undergraduate students
Source: J Med Libr Assoc. 2019 Oct 1;107(4):572–8. doi: 10.5195/jmla.2019.663 (PMC6774555; doi:10.5195/jmla.2019.663)
Supplement: Appendix A [file jmla-107-572-s001.pdf]

JMLA Journal of the Medical Library Association

SC

Supplemental content to
J Med Libr Assoc. Oct;107(4):dx.doi.org/10.5195/jmla.2019.663
www.jmla.mlanet.org
© McGowan 2019

# Reimagining information literacy instruction in an evidence-based practice nursing course for undergraduate students

Bethany Sheriese McGowan

## APPENDIX A

### Storyboard for the design of an in-class search activity

<table>
<tr><td>Name: Evidence-Based Practice (EBP) Course Redesign</td><td>Title: Create a search strategy</td><td>Date of production: January 23, 2017</td></tr>
<tr><td colspan="2" rowspan="2">Description of activity: “Searching as Strategic Exploration: Building Block Activity”<br><br>This hands-on activity will complement a lecture on search building, which includes teaching the use of Boolean operators; selection of keywords, natural language, and controlled vocabulary; use of wildcards; and selection of appropriate databases.</td><td>Action details & notes<br><br>Record a closed-caption video lecture on how to build a basic search in both PubMed and CINAHL. Provide video in advance of class, as a pre-work assignment.<br><br>The search examples will be based on a problem, intervention, comparison, outcome (PICO) question.</td></tr>
<tr><td>Maps to:<br><br>Unit 2 (Weeks 3–6): Information literacy and quantitative literacy, building search strategies<br><br>Redesign objective 2: Students will participate and engage in in-class activities</td></tr>
<tr><td colspan="3">Programming notes<br><br>Embed lecture video in Blackboard and track views. Use Camtasia or PowerPoint to embed quiz questions into lecture videos and have students submit answers to quiz questions as homework.</td></tr>
</table>
