# Supplementary material for: Reimagining information literacy instruction in an evidence-based practice nursing course for undergraduate students
Source: J Med Libr Assoc. 2019 Oct 1;107(4):572–8. doi: 10.5195/jmla.2019.663 (PMC6774555; doi:10.5195/jmla.2019.663)
Supplement: Appendix B [file jmla-107-572-s002.pdf]

JMLA Journal of the Medical Library Association

Supplemental content to
J Med Libr Assoc. Oct;107(4):dx.doi.org/10.5195/jmla.2019.663
www.jmla.mlanet.org
© McGowan 2019

Reimagining information literacy instruction in an evidence-based practice nursing course for undergraduate students

Bethany Sheriese McGowan

APPENDIX B

Sample course schedule

Unit	Course objectives	Class activities	Assignments
Unit 1 (Weeks 1–2): course introduction		Syllabus, textbook, and rubric review<br>Time management and effective learning techniques with learning styles inventory (in-class survey)<br>American Psychological Association (APA) writing style overview<br>Download Zotero and Word plug-ins on personal computers<br>Data visualization: creating graphs with Excel and Tableau<br>Declare group research topic selection<br>Declare individual research topic selection<br>Twitter data analysis, group project: samples from previous semesters	Required readings<br>Supplementary course materials: assigned quizzes, videos, and recorded lectures<br>Choose 4–6 group members and consider group project topic, consider using GroupMe<br>Consider an individual research topic<br>Utilize Melnyk's Critically Appraising Evidence resources: general appraisal tools, validity/reliability checklist, evaluation tables

JMLA Journal of the Medical Library Association

Supplemental content to
J Med Libr Assoc. Oct;107(4):dx.doi.org/10.5195/jmla.2019.663
www.jmla.mlanet.org
© McGowan 2019

Unit	Course objectives	Class activities	Assignments
Unit 2 (Weeks 3–6): information literacy and quantitative literacy	Describe the principles of research and the process of EBP in relation to health care<br>Use information and information technologies ethically, legally, and proficiently according to the Association of College & Research Libraries (ACRL) Framework for Information Literacy for Higher Education<br>Explain the purpose and methodology of various types of quantitative and qualitative research design in health care<br>Examine economic, ethical, and legal issues related to conducting research	Lectures and in-class activities<br>Information literacy: selecting databases; building search strategies; locating, retrieving, and organizing research articles; building literature review tables; preparing a reference list; synthesizing literature<br>Quantitative literacy: validity and reliability; interpretation and critique of quantitative data; intro to SafeRx database; ethical reasoning<br>Group research project: refine search strategies, create a PRISMA flow diagram	Required textbook and journal article readings<br>Follow directions for Twitter group project in Instruction Manual<br>View videos and other supplementary materials in Blackboard<br>Complete National Institutes of Health (NIH) certification: Completion for Protecting Human Research Participants<br>Individual research project: develop a research question and search strategy, select appropriate databases, retrieve and organize research articles
Unit 3 (Weeks 7–16): deliverables	Evaluate the quality of research evidence to determine scientific merit, strengths, and limitations relevant to clinical practice.<br>Discuss the process of translating research evidence into practice<br>Demonstrate the characteristics of an innovator that are necessary for evidence-based practice (EBP), which include leadership, a sense of inquiry, flexibility to change, awareness of self and of the environment, effective communication, critical thinking, lifelong learning, and professionalism	Complete Twitter data analysis group project<br>Complete and present individual research project<br>Complete and present group project: finalize poster project for undergraduate research fair	Required textbook and journal article readings<br>View videos and other supplementary materials in Blackboard<br>Complete Twitter trawl project<br>Complete group project: locate and retrieve articles; evaluate, organize, and summarize research findings; oral presentation in class<br>Complete individual project: finalize poster project for undergraduate research fair
