# Supplementary material for: Reimagining information literacy instruction in an evidence-based practice nursing course for undergraduate students
Source: J Med Libr Assoc. 2019 Oct 1;107(4):572–8. doi: 10.5195/jmla.2019.663 (PMC6774555; doi:10.5195/jmla.2019.663)
Supplement: Appendix C [file jmla-107-572-s003.pdf]

JMLA Journal of the Medical Library Association

SC
Supplemental content to
J Med Libr Assoc. Oct;107(4):dx.doi.org/10.5195/jmla.2019.663
www.jmla.mlanet.org
© McGowan 2019

Reimagining information literacy instruction in an evidence-based practice nursing course for undergraduate students

Bethany Sheriese McGowan

APPENDIX C

Evidence-based practice course student confidence survey

	Strongly disagree	Disagree	Somewhat disagree	Neither agree nor disagree	Somewhat agree	Agree	Strongly agree
I feel confident in my ability to select appropriate databases when looking for information.							
I feel confident in my ability to build a search that will help me find the information I need (e.g., create a problem, intervention, comparison, outcome, time [PICOT] question, select appropriate keywords, set inclusion criteria)							
I feel confident in my ability to retrieve the full text for article citations.							
I feel confident in my ability to share and explain how I retrieved search results.							

JMLA Journal of the Medical Library Association

SC
Supplemental content to
J Med Libr Assoc. Oct;107(4):dx.doi.org/10.5195/jmla.2019.663
www.jmla.mlanet.org
© McGowan 2019

	Strongly disagree	Disagree	Somewhat disagree	Neither agree nor disagree	Somewhat agree	Agree	Strongly agree
I feel confident in my ability to use American Psychological Association (APA) style formatting when writing research papers.							
I feel confident in my ability to use citation management tools, like Zotero, to organize citations and remove duplicates.							
I feel confident in my ability to evaluate the quality of research evidence to determine scientific merit, strengths, and limitations.							
